# Focused Workshop

**DOI:** 10.1111/ene.70675

**Published:** 2026-06-26

## Saturday, June 27 2026

## AI in hospital neurology

## FW01_1

### AI in acute stroke

#### S. Wegener

##### University Hospital Zurich and University of Zurich, Switzerland

## FW01_2

### AI in disorders of consciousness

#### A. Estraneo

##### IRCCS, Don Gnocchi Foundation, Florence, Italy

## FW01_3

### AI in identification & diagnosis of multiple sclerosis

#### C. Oreja‐Guevara

##### Hospital Clinico San Carlos, Madrid, Spain

## EAN/EPNS: How to deal with cerebrovascular diseases in children and adolescents

## FW02_1

### Stroke across pediatric age

#### I. Kopyta

##### Department of Child Neurology, Medical University of Silesiat, Katowice, Poland

Stroke is a rare condition in the pediatric population, but it can affect children of all ages, most commonly ‐newborns and young infants. Neonatal stroke is excluded from the definition of pediatric arterial ischemic stroke due to its specific risk factors and prognosis. The diagnosis of PAIS (Pediatric arterial ischemic stroke) can be made in the case of sudden neurological symptoms, at age between 29 days and 18 years, with vascular pathology detected by neuroimaging, correspond in its location to clinical symptoms. The incidence of PAIS is estimated at 3–13/100,000 per year; the risk factors are arteriopathies (acquired and congenital, with a progressive, stationary or remitting course), heart diseases and thrombophilia. Confirmation of PAIS is based on the results of a neuroimaging and the method of choice is magnetic resonance imaging with MRA of the cerebral and neck vessels. If the probable delay in MRI performance exceeds 25 min, then CT imaging should be performed. A child diagnosed with PAIS is currently a potential candidate for thrombolytic therapy and thrombectomy. Antiplatelet therapy is an alternative in the case of exceeding the therapeutic window and is a method used in secondary prevention. Most children with PAIS will present the permanent neurological deficits; approximately 3% of children with PAIS die during the acute phase of the disease. A key issue in improving prognosis and increasing access to modern therapeutic methods for children with PAIS is improving awareness on PAIS.

**Disclosure:** Nothing to disclose.

## FW02_2

### Arteriopathies in pediatric stroke

#### R. Tudorache

##### Pediatric Neurology Discipline, Neuroscience Department, “Carol Davila” University of Medicine and Pharmacy, Bucharest, Romania, and Université Paris Cité, France

Pediatric arterial ischemic stroke (PAIS) has been associated with a wide range of underlying conditions. In developed countries, the most common etiologies include cerebral arteriopathies, congenital or acquired heart diseases, and severe systemic infections. Cerebral arteriopathy is a major contributor to PAIS, accounting for nearly half of all cases and significantly increasing the risk of both initial and recurrent stroke. These arteriopathies may be congenital, reflecting abnormal vascular development, or acquired as a result of disruption of vascular homeostasis. The term encompasses a broad spectrum of vascular imaging abnormalities, including cervicocephalic arterial dissection, moyamoya, vasculitis, post‐varicella angiopathy, and idiopathic focal cerebral arteriopathy. Their classification has undergone several revisions over the past two decades. Among intracranial arteriopathies, focal cerebral arteriopathy (FCA) is the most frequently identified subtype, accounting for up to 25% of cases. FCA is a dynamic entity that may exhibit progression or relapse and is characterized by inflammatory arterial stenosis, often developing secondary to infection, particularly viral infections such as upper respiratory tract infections or varicella. The vascular involvement typically affects the carotid T, especially the proximal segment of the middle cerebral artery. Diagnosis relies on clinical presentation and neuroimaging. Brain MRI is the first‐line imaging modality in pediatric stroke, enabling early differentiation from stroke mimics, while in cases of suspected FCA, vessel wall imaging is particularly useful. Therapeutic approaches remain heterogeneous. Antithrombotic therapy is most commonly used for secondary stroke prevention, while corticosteroids have been proposed to limit disease progression, with several studies currently ongoing.

**Disclosure:** Nothing to disclose.

## FW02_3

### Hyperacute treatment for pediatric stroke (IVT, EVT)

#### P. Sporns

##### Stadtspital Zürich, Diagnostic and Interventional Neuroradiology, Switzerland

## Timely evaluation of a first‐time seizure

## FW03_1

### Timely evaluation of a fist seizure ‐ insights from the SWISS FIRST Study

#### B. Jin

##### Sleep‐Wake‐Epilepsy‐Center, Department of Neurology, Inselspital, Bern University Hospital, University of Bern, Bern, Switzerland

Early diagnosis of a first unprovoked epileptic seizure and estimation of seizure recurrence risk is challenging and important to decide about initiating antiseizure medication. In SWISS FIRST, a prospective, observational, multicenter study across seven hospitals in Switzerland, we assessed adult patients suspected for first unprovoked epileptic seizure and their development over two years. We determined diagnostic accuracy of baseline EEG and MRI based on their diagnosis two years after first event. We developed a binary logistic regression model for seizure recurrences on the baseline EEG and MRI and clinical characteristics of the patients.

We recruited 636 patients with suspected first unprovoked epileptic seizure. 238 were finally diagnosed with epilepsy. Sensitivity for epileptiform activity in EEG was 31.5%, specificity was 97.3%. Potential epileptogenic lesions were present in 40.3% amongst epilepsy patients, but also abundandly in patients with only a first unprovoked seizure (31%) and non‐epileptic seizure patients (28.4%). Patients with sleep‐related seizures (OR 2.01, 95% CI 1.17‐3.45; *p* = 0.01), motor seizures (OR 2.39, 95% CI 1.57–3.66; *p* < 0.001), seizures with impaired consciousness (OR 1.6, 95% CI 1.05 to 2.46; *p* = 0.03), focal epileptiform activity (OR 1.87, 95% CI 1.08–3.2 *p* = 0.02), presence of potential epileptic lesion (OR 2.49, 95% CI 1.25–5.08; *p* = 0.01), and diffusion restriction (OR 2.78, 95% CI 1.54–5.04; *p* < 0.001) were associated with increased seizure recurrence risk.

These findings of the SWISS FIRST study are expected to influence future patient management and therapy guidelines after first unprovoked epileptic seizure.

**Figure:**
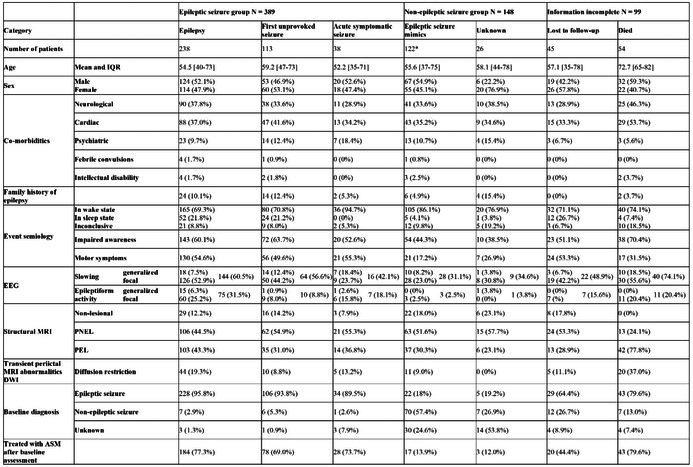
**FIGURE 1** Clinical characteristics, baseline EEG and MRI findings.

**Figure:**
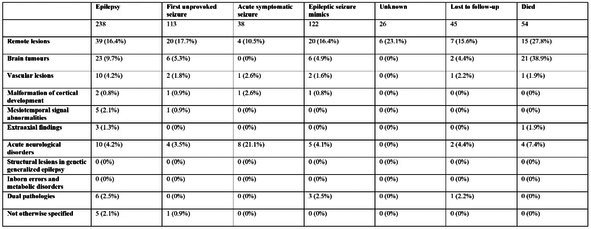
**FIGURE 2** Specification of the potentially structural epileptogenic lesions.

**Figure:**
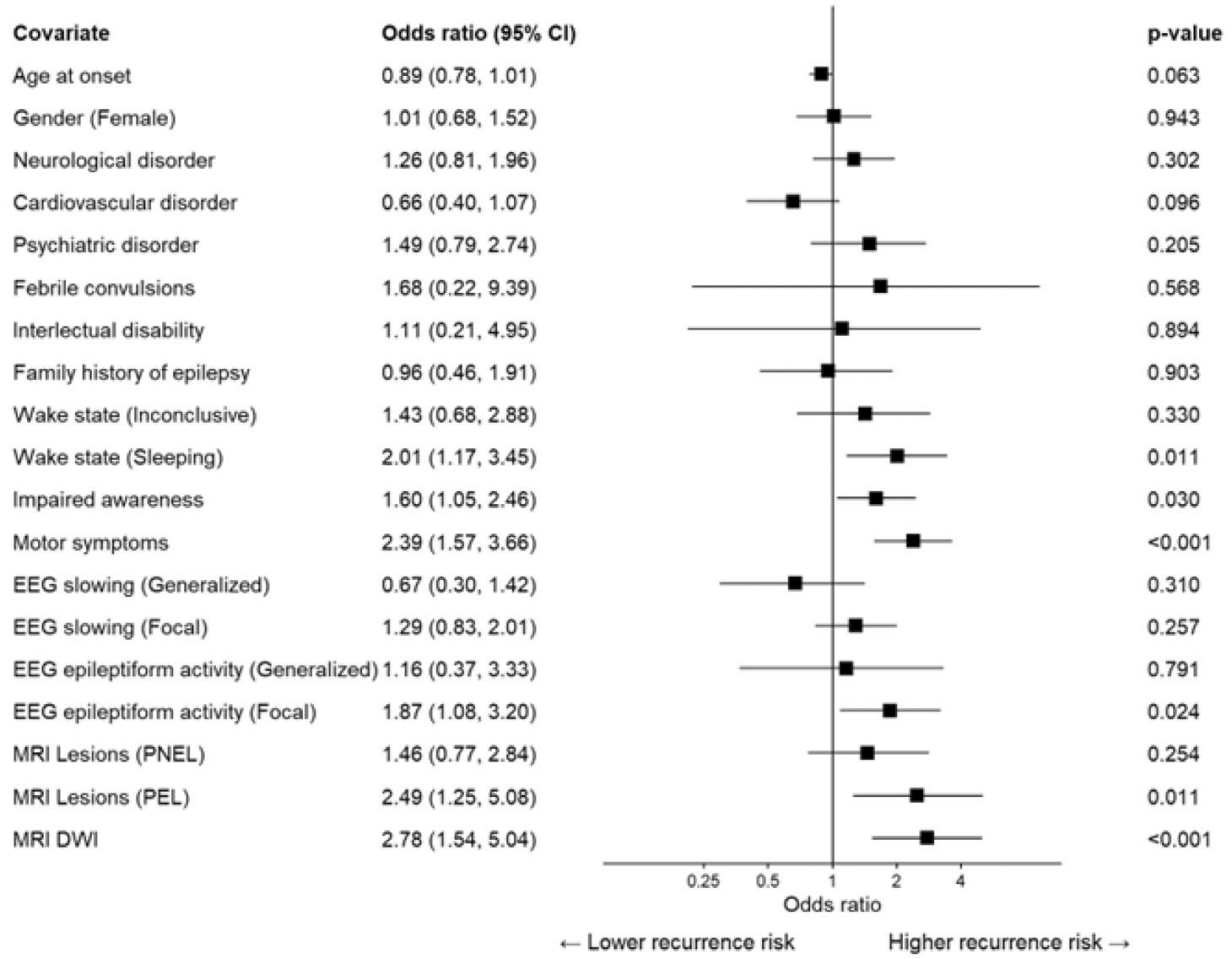
**FIGURE 3** Multivariable logistic regression model for seizure recurrences.

**Disclosure:** Nothing to disclose.

## FW03_2

### Timely evaluation of a first‐time seizure – the need for seizure units

#### M. Seeck

##### Hopitaux Universitaires de Genève‐HUG, Department of Neurology, Geneva, Switzerland

## FW03_3

### Timely evaluation of a first‐time seizure – how to fill the gaps?

#### T. Marson

##### The Walton Centre NHS Foundation Trust, Unit of Neuroscience Research, Liverpool, United Kingdom

## EAN/IFCN‐EMEAC: New developments in diagnostics and non‐invasive therapy in cognitive disorders

## FW04_1

### TMS‐EMG for differential diagnosis of dementias

#### A. Benussi

##### University of Trieste, Italy

## FW04_2

### Hyperexcitability in the pathogenesis of Alzheimer's disease and transcranial electrical stimulation for the treatment

#### A. Kamondi

##### Semmelweis University, Budapest, Hungary

## FW04_3

### Repetitive TMS of the precuneus for the treatment in AD

#### A. Antal

##### Department of Neurology, UMG, Göttingen, Germany

Non‐invasive Brain Stimulation (NiBS) techniques such as repetitive Transcranial Magnetic Stimulation (rTMS) and transcranial Electrical Stimulation (tES), including Direct Current and Alternating Current Stimulation (tDCS, tACS), allow to modulate neuronal excitability, oscillatory activity and to boost cortical functions, hereby possibly offering a therapeutic potential to slow down cognitive decline. Cognitive decline reaches medical attention for about 8–25% of individuals aged over 65, when suffering from Mild Cognitive Impairment (MCI). Around 15% of MCI patients develop dementia within two years, supporting a medical need for early diagnosis and treatment. Recent investigations propose rTMS of the precuneus as a potential therapeutic tool to improve network activity and cognitive functions in older individuals with cognitive impairments. However, several subject‐specific factors, for example, anatomical and physiological differences, gender, brain state before and during stimulation, and also methodological factors, such as the type of the task applied in combination with the stimulation, can confound the effects of stimulation. This talk aims to provide an overview of the commonly utilized rTMS protocols, elucidating their individual merits and drawbacks.

**Disclosure:** Andrea Antal has nothing to disclose with regard to this talk. AA is a vice president of the European Society for Brain Stimulation, Member‐at‐Large at the EMEAC – IFCN Member of the safety or advisory boards: Boss‐Stroke, Brady, Brainn, MemoSlap, GifteD, DisCoVer, PlatoScience Paid consultant at NeuroConn, Ilmenau, and she is a paid advisor at Electromedical Products International (Pulvinar), USA. Member of the advisory board at PlatoScience Paid Editor by Elsevier Supported by the DFG (AN 687/9‐1), EU‐Horizon 2020 (PAINLESS, No. 101057367), BMBF (NEURO VR‐Plus 16SV9248), DAAD‐PPP (57702891).

## The neurological face of menopause: Rethinking migraine, stroke and Parkinson's in postmenopausal women

## FW05_1

### Menopause and migraine

#### A. Szewczyk

##### Medical University of Lublin, Department of Neurology, Lublin, Poland

## FW05_2

### Menopause and stroke

#### D. Aguiar de Sousa

##### Hospital de Santa Maria ‐ Faculdade de Medicina de Lisboa, Lisbon, Portugal

## FW05_3

### Menopause and Parkinson's disease

#### K. Rukavina

##### Berlin, Germany

## The cerebellum beyond motor function: Uncovering cognitive‐affective roles

## FW06_1

### The 3 facets of the cerebellar syndrome: Emphasis on the cerebellar cognitive affective syndrome

#### M. Manto

##### Brussels, Belgium

## FW06_2

### The role of the cerebellum in emotions

#### D. Timmann

##### Department of Neurology and Center for Translational Neuro‐ and Behavioral Sciences (C‐TNBS), Essen University Hospital, University of Duisburg‐Essen, Essen, Germany

The link between the cerebellum and emotional behavior will be discussed, beginning with an overview of known anatomical connections between the cerebellum and limbic structures, including the amygdala, PAG, hippocampus, and prefrontal cortex. Second, early animal studies demonstrating the involvement of the cerebellum in autonomic functions related to the expression of emotions will be summarized, including blood circulation and respiration. Third, functional brain imaging studies in healthy human participants examining emotional tasks will be reviewed, along with observations of altered emotional control in patients with cerebellar disorders, including degenerative ataxias and focal lesions. These findings will be complemented by structural and functional imaging evidence of disrupted cerebello–limbic interactions in patients with neuropsychiatric disorders, in particular anxiety disorders.

Finally, we will focus on fear, an important emotion for survival. The cerebellum contributes not only to innate affective and defensive behaviors but also to learned fear responses. Early rodent lesion studies showed that fear‐conditioned bradycardia is markedly reduced after vermal lesions. To date, only a few studies have examined fear conditioning in patients with cerebellar disease, in which behavioral deficits appear to be milder. Most evidence for cerebellar involvement in human fear conditioning comes from neuroimaging studies in healthy participants. Threat‐prediction–related activations extend beyond the vermis, with prominent involvement of the lateral cerebellum. There is initial evidence that the cerebellum contributes to prediction‐error signals driving fear extinction via interactions with the VTA. These findings demonstrate that cerebellar control extends beyond autonomic functions and includes higher‐order processes such as reward processing.

**Disclosure:** Dagmar Timmann received funding from the German Research Foundation (project number 316803389 – SFB1280), the European Union's Horizon 2020 research and innovation program under the Marie Skłodowska‐Curie grant agreement No 956414, and the Bernd Fink Foundation.

## FW06_3

### A functional atlas of the cerebellum

#### C. Nettekoven

##### Department of Experimental Psychology, University of Oxford, Oxford, UK

Once considered primarily a motor structure, the cerebellum is now known to contribute to cognitive processes. Cerebellar damage can therefore produce cognitive disturbances in addition to motor deficits. However, limited knowledge of cerebellar functional organization continues to constrain the interpretation of cerebellar involvement in neurological disease.

This limitation largely reflects the absence of a comprehensive functional map of the human cerebellum. Although different cerebellar regions are engaged during motor, cognitive, and emotional tasks, these regions remain difficult to reliably delineate using existing atlases, particularly in the presence of substantial inter‐individual variability.

In this talk, I will present the first comprehensive functional atlas of the human cerebellum, derived by integrating multiple large‐scale functional MRI datasets spanning a wide range of motor, cognitive, and language tasks. Using a data‐driven modelling approach, we identified distinct cerebellar regions with characteristic functional profiles and connectivity patterns. The atlas reveals a clear functional topography, with anterior cerebellar regions predominantly involved in motor control, posterior regions, particularly lobule VIIA (Crus I and II)—engaged in higher‐order cognitive processes.

Importantly, the atlas enables precise mapping of cerebellar function at the individual level, addressing the limitations of group‐based maps. I will discuss how this framework improves our ability to interpret cerebellar activation and lesion effects, and how it can inform the study of cognitive and affective symptoms in neurological disorders along with potentially improve surgical planning. By providing a unifying functional reference, this work supports a more integrated view of the cerebellum as a key contributor to both motor and non‐motor brain function.

**Figure:**
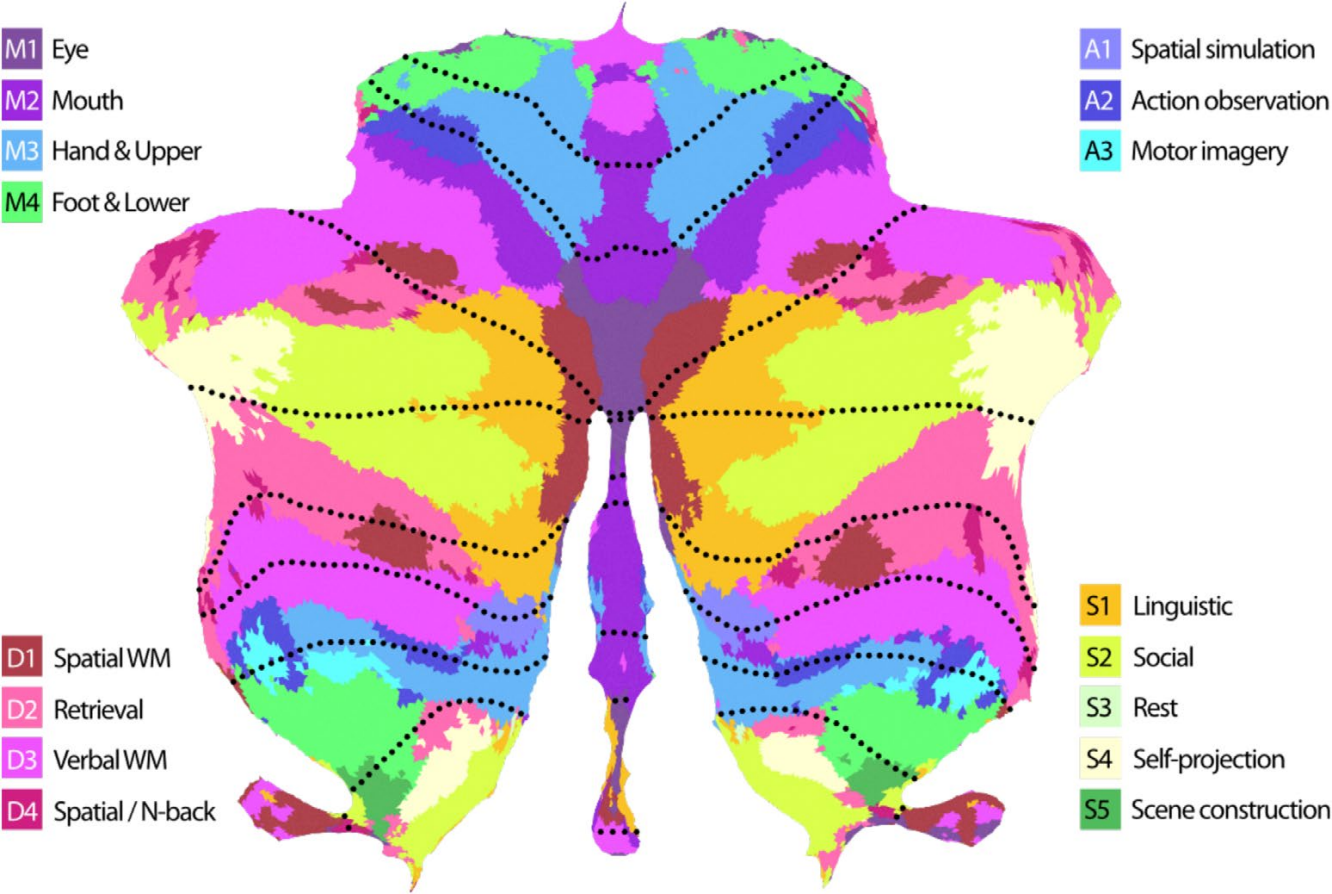
**FIGURE 1** Cerebellar functional atlas. Symmetric atlas at medium level of granularity. The labels of each region consists of a capital for the domain (M, A, D, S), a number for region (1–4), and a lower‐ case letter for the subregion (a–d).

**Disclosure:** Nothing to disclosure.

## Sunday, June 28 2026

## AI in outpatient neurology

## FW07_1

### AI in epilepsy

#### S. Beniczky

##### Epilepsy Hospital Filadelfia, Danish Epilepsy Centre ‐ Department of Clinical Neurophysiology, Dianalund, Denmark

## FW07_2

### AI for Parkinson's disease and rare parkinsonian conditions

#### C. Lambert

##### National Hospital for Neurology and Neurosurgery/UCL, Functional Imaging Laboratory, UCL Department of Imaging Neuroscience, London, United Kingdom

## FW07_3

### AI in headache care

#### A. Stubberud

##### NorHead Norwegian Centre for Headache Research, Department of Neuromedicine and Movement Science, NTNU Norwegian University of Science and Technology

The use of artificial intelligence (AI) in headache care and research is rapidly expanding. AI has the potential to enhance clinical task such as diagnosis, treatment selection and prediction of disease trajectories. To date, several key areas have received the most attention in the headache literature. First, large language models have been used for automated extraction of patient‐level data, as well as to support the training of healthcare providers and patient education. Second, a variety of AI models have been used to diagnose and classify headache disorders using both clinical and paraclinical data. There are also some indications that such models could improve diagnostic accuracy among non‐neurologists. Third, AI models have shown promise in optimizing treatment selection and predicting treatment responses in primary headache disorders. Finally, predictive models that can forecast day‐to‐day headache occurrence, as well as longer‐term disease trajectories, show encouraging results. While the current body of literature demonstrates promising developments, there remains a substantial need for external validation and clinical trials to enable the translation of these models into clinically applicable tools.

**Disclosure:** AS has received speaker honoraria from TEVA. He is co‐founder and shareholder of Nordic Brain Tech AS and hold a patent related to the Cerebri biofeedback treatment for migraine. He is junior editor for Cephalalgia.

## EAN/MDS‐ES: Current role and future perspectives of neuromodulation in the treatment of Parkinson's disease and dementia with Lewy bodies

## FW08_1

### Current role and future perspectives of deep bran stimulation in the treatment of PD

#### E. Moro

##### CHU de Grenoble, Division of Neurology, Department of Psychiatry and Neurological Rehabilitation, Grenoble, France

## FW08_2

### Focused ultrasound brain therapy is a new tool in the box

#### G. Deuschl

##### Universitätsklinikum Schleswig Holstein Campus Kiel, Department of Neurology, Kiel, Germany

## FW08_3

### Potential of non‐invasive brain stimulation (NIBS) for the treatment of PD and DLB

#### I. Rektorova

##### Mararyk University, Brno Teaching Hospital Svata Anna, 1st Department of Neurology, Brno, Czech Republic

## EAN/EANS: Spontaneous intracranial hypotension: The headache neglected by neurologist

## FW09_1

### Clinical spectrum and imaging essentials in Spontaneous Intracranial Hypotension

#### T. Dobrocky

##### Diagnostic and Interventional Neuroradiology, University of Bern, Inselspital, Bern, Switzerland

Spontaneous intracranial hypotension (SIH) is an important and frequently underrecognized cause of secondary headache with a broad and sometimes misleading clinical spectrum. Although orthostatic headache is the classic presentation, patients may develop atypical symptoms including non‐orthostatic headache, cranial nerve palsies, cognitive and behavioral changes, movement disorders, or impaired consciousness, often leading to delayed diagnosis. Over the past years, substantial progress has been made in elucidating the underlying etiologies of SIH, particularly distinct types of spinal cerebrospinal fluid (CSF) leaks and CSF–venous fistulas. In parallel, standardized diagnostic algorithms integrating brain MRI and advanced spinal imaging have been established, transforming SIH into a well‐defined and treatable condition. Neuroimaging is central to diagnosis and management, with characteristic brain MRI findings and targeted spinal techniques such as dynamic myelography enabling precise leak localization and guiding therapy. This session reviews the clinical spectrum of SIH and highlights imaging essentials for accurate diagnosis and effective treatment.

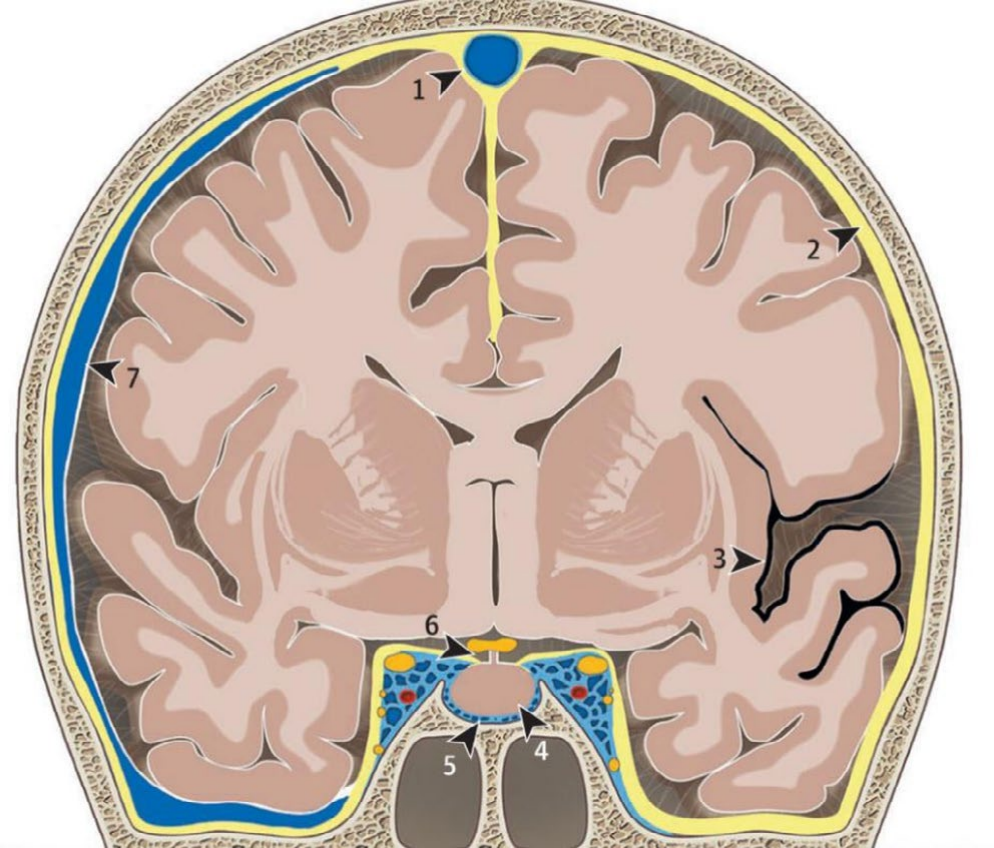

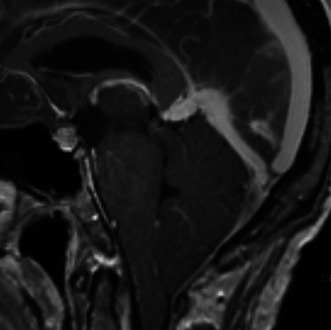

**Disclosure:** No disclosures.

## FW09_2

### Blood patching and percutaneous therapy for spontaneous intracranial hypotension

#### L. Carlton Jones

##### Guys and St Thomas Hospital, Department of Radiology, Westminster Bridge Road, London, United Kingdom

## FW09_3

### Surgical therapy, outcome and long‐term complications in spontaneous intracranial hypotension

#### J. Beck

##### Universitätsklinikum Freiburg, Neurosurgery, Freiburg, Germany

## Technologies to identify and preserve residual brain functions in the ICU

## FW10_1

### TMS‐EEG to probe brain networks in unconsciousness

#### S. Casarotto

##### Dept. Biomedical and Clinical Sciences, University of Milan; IRCCS Fondazione Don Carlo Gnocchi ONLUS, Milan

**Introduction:** Attributing consciousness to others always implies an inferential process, which is particularly challenging when evaluating patients who remain in prolonged unresponsive wakefulness syndrome (UWS) after severe brain injury. Behavioral evaluations alone may be misleading due to impaired sensory‐motor functions. Neural responses to verbal commands can indicate intentional processing, but require intact language, executive functions, and sustained attention. Auditory paradigms with increasing complexity may reveal residual perceptual awareness without requiring active participation, though they still depend on attention and preserved sensory pathways. Measures of intrinsic brain activity, such as perturbational complexity, can inform about capacity for consciousness even when sensory‐motor channels are compromised.

**Methods:** This study applied a multilayer neurophysiological assessment in 16 UWS patients hospitalized in an Intensive Rehabilitation Unit.

**Results:** Twelve patients recovered at least minimal behavioral signs of responsiveness. Before this occurred, intrinsic brain activity suggested a capacity for consciousness in all these patients, 25% of whom also showed neural responses to verbal commands and 8% perceptual activations. Clinical evoked potentials revealed significant sensory‐motor impairments in most patients, likely contributing to negative findings of behavioral, task‐based and perceptual evaluation.

**Discussion:** These results demonstrate feasibility and sensitivity of multilayer neurophysiological assessment in subacute rehabilitation. This approach is necessary to evaluate residual consciousness beyond behavioral responsiveness, progressively moving from task‐based modulation of brain activity to perceptual awareness to intrinsic brain dynamics. The accurate detection of covert capacity for consciousness is crucial to guide treatment, since 75% of patients who remain in prolonged UWS may show late recovery.

**Disclosure:** I am currently advisor and share‐holder of Intrinsic Powers, a spin‐off of the University of Milan.

## FW10_2

### Pupillometry and functional NIRS to identify covert consciousness in the ICU

#### D. Kondziella

##### Copenhagen University Hospital – Rigshospitalet, Department of Neurology, Copenhagen, Denmark

## FW10_3

### Multimodal neuromonitoring to prevent secondary brain injury in the ICU

#### R. Helbok

##### Johannes Kepler University Linz, Department of Neurology, Linz, Austria

## Monday, June 28 2026

## EAN/MDS‐ES: Repurposing GLP‐1 receptor agonists: New frontiers for neuroprotection in neurological diseases

## FW12_1

### Beyond glycemic control: Uncovering the neuroprotective role of GLP‐1 receptor agonists

#### F. Ferrari

##### Department of Health Sciences, Università del Piemonte Orientale, Novara Italy

**Introduction:** Glucagon‐like peptide‐1 receptor agonists (GLP‐1 RAs), developed for type 2 diabetes mellitus and obesity, are emerging as neuroprotective candidates in neurological disorders [1,2]. This lecture provides a mechanistic overview of their central nervous system (CNS) actions.

**Methods:** Literature review focused on GLP‐1 RAs pharmacology, intracellular signaling and downstream pathways relevant to neuroprotection.

**Results:** GLP‐1 receptors are broadly expressed in CNS and their activation engages cyclic AMP‐dependent and phosphoinositide 3‐kinase signaling, producing pleiotropic effects [3,4]: (i) attenuation of neuroinflammation via microglial polarization to anti‐inflammatory phenotype, NFkB and NLRP3 inflammasome inhibition, suppression of TNF‐alpha, IL‐1‐beta and IL‐6, and prevention of neurotoxic reactive astrocyte conversion; (ii) anti‐apoptotic activity through Bcl‐2 upregulation and Bax/caspase‐3 suppression; (iii) mitochondrial biogenesis via PGC‐1‐alpha and sirtuin‐1, with reduction of oxidative stress; (iv) neurotrophic and synaptic support through brain‐derived neurotrophic factor/TrkB signaling, AMPA receptor trafficking, postsynaptic density protein‐95 upregulation, hippocampal neurogenesis and long‐term potentiation; (v) proteostasis restoration via ULK1‐dependent autophagy, reducing amyloid‐beta, hyperphosphorylated tau and alfa‐synuclein aggregates; (vi) preservation of blood‐brain barrier and neurovascular unit. Additional body‐to‐brain signaling via vagal afferents contributes to indirect central neuroprotection.

**Conclusion:** GLP‐1 RAs display multifaceted, converging neuroprotective mechanisms targeting the triad of neurodegeneration: neuroinflammation, mitochondrial dysfunction and proteinopathy. Their established safety profile and pleiotropy make them strong candidates for repurposing in neurology.

**References:** [1] Ferrari et al. (2022). Pharmacol Ther. 239:108277; [2] Ferrari et al. (2020). Pharmacol Res. 160:105018; [3] Ros‐Madrid et al. (2025). Can J Physiol Pharmacol. 103:369–377; [4] Roy et al. (2025). Neurotherapeutics. 22:e00712.

**Disclosure:** The author declares no conflicts of interest.

## FW12_2

### GLP‐1 receptor agonists in neurodegenerative diseases: What we have learned so far?

#### D. Athauda

##### University College London, Sobell Department of Motor Neuroscience and Movement Disorders, London, United Kingdom

## FW12_3

### GLP‐1 Receptor agonists for the treatment and prevention of stroke

#### E. Vercalsteren

##### Karolinska Institute, Department of Clinical Science and Education, Stockholm, Sweden

## EAN/EFIC: Revised guidelines for neuropathic pain: Diagnostic tests, pharmacotherapy and neuromodulation

## FW13_1

### How to diagnose neuropathic pain. Evidence‐based recommendations

#### A. Truini

##### University of Rome “La Sapienza”, Department of Human Neuroscience, Roma, Italy

## FW13_2

### Revised Recommendations on the use of pharmacological therapies for neuropathic pain relief

#### X. Moisset

##### Université Clermont Auvergne, CHU Clermont‐Ferrand, Inserm, Neuro‐Dol, Clermont‐Ferrand, France

Neuropathic Pain remains a major clinical challenge, with many patients experiencing insufficient pain relief and treatment‐related adverse effects. The Neuropathic Pain Special Interest Group undertook a comprehensive update of its treatment recommendations, incorporating new randomized controlled trials and advances in evidence synthesis.

In this presentation, we will summarize the pharmacological findings of a large systematic review and meta‐analysis including double‐blind, randomized, placebo‐controlled trials published up to February 2024. Across 284 pharmacological trials involving tens of thousands of participants, treatments were evaluated using standardized efficacy and safety outcomes, including responder rates, numbers needed to treat (NNT), and numbers needed to harm (NNH), with evidence certainty assessed using the GRADE framework.

Participants will gain an updated overview of evidence‐based pharmacological strategies for neuropathic pain. Strong recommendations support the use of tricyclic antidepressants, α2δ‐ligands (gabapentinoids), and serotonin–noradrenaline reuptake inhibitors as first‐line treatments. Second‐line options include topical therapies such as high‐concentration capsaicin patches, capsaicin cream, and lidocaine 5% plasters. Third‐line strategies include botulinum toxin A and opioids in selected situations. The presentation will also highlight the magnitude of treatment effects, safety considerations, and how these therapies compare in terms of benefit–risk balance.

Beyond summarizing the evidence, the session will provide practical guidance on how to translate these updated recommendations into everyday clinical decision‐making. Attendees will leave with a clear understanding of current pharmacological treatment hierarchies for neuropathic pain and the strengths and limitations of the available evidence.

**Disclosure:** Funding by NeuPSIG (IASP), ERANET‐Neuron and Dr Jennie Gwynn Legacy Fund.

## FW13_3

### New recommendations for the use of neuromodulation to relieve neuropathic pain

#### N. Attal

##### Hôpital Ambroise Paré, Department of Pain Treatment, Boulogne‐Billancourt, France

## EAN/EFAS: Digital dysautonomia: The role of wearables and AI revolution

## FW14_1

### Role of wearables and remote monitoring in assessing autonomic nervous system parameters

#### C. Falup Pecurariu

##### Transilvania University Brasov, Department of Neurology, Brasov, Romania

## FW14_2

### AI in the autonomic area

#### W. Struhal

##### Universitätsklinik Tulln, Department of Neurology, Tulln, Austria

## FW14_3

### Personalized assessment in autonomic dysfunction – role of digital biomarkers

#### A. Fanciulli

##### Innsbruck Medical University, Department of Neurology, Innsbruck, Austria

## Tuesday, June 30 2026

## Understanding Alzheimer's disease: Disease models and diagnostic criteria

## FW15_1

### The clinical context of treatment and prevention of Alzheimer's disease

#### K. Frederiksen

##### Copenhagen University Hospital – Rigshospitalet, Memory Disorders Research Unit, Copenhagen, Denmark

## FW15_2

### The case in favour of the Alzheimer Association criteria and Alzheimer's as a biological condition

#### C. Teunissen

##### Neurochemistry Lab, Amsterdam, The Netherlands

## FW15_3

### International Working Group criteria and the clinical eco system they come with

#### G. Frisoni

##### Hôpitaux Universitaires de Genève, Geneva, Switzerland

## Neurorehabilitation goes digital – management of cognitive and behavioral deficits

## FW16_1

### Brain computer interface for post‐coma patients

#### E. Molteni

##### University College London, Institute of Health Informatics, London, United Kingdom

## FW16_2

### Enhancing cognitive neurorehabilitation through virtual reality

#### A. Sokolov

##### Centre Hospitalier Universitaire Vaudoise ‐ CHUV BH07, Department of Neurology, Lausanne, Switzerland

## FW16_3

### Digital solutions for diagnostics and treatment of psychiatric disorders in neurorehabilitation

#### K. Rauen

##### Institute of Emergency Medicine, University Hospital Zurich & Neuroscience Center Zurich, University of Zurich, Switzerland

Digital solutions are increasingly accepted as add‐on tools for diagnostics and treatment in patients and their caregivers. Despite rapid developments and rising approvals only 39% of Digital Health Applications target psychiatric disorders and none of them addresses psychiatric burden in neurological patients during neurorehabilitation. Today, most digital solutions target depression (27%), anxiety (23%), insomnia (10%), cognition (7%), and various psychiatric conditions. It is well known that neurological patients after stroke or traumatic brain injury (TBI) particularly suffer from brain‐injury induced depression, cognitive dysfunction and/or sleep disorders with raising evidence that brain‐injury induced affective disorders differ in their pathophysiology and clinical presentation from Major Depressive Disorder (MDD). Consecutively, current digital solutions may not adequately address neurological patients’ needs and often require caregivers’ support. Therefore, it is time to close the gap on brain‐injury specific Digital Health Applications for patients during and beyond neurorehabilitation aiming for better neuropsychiatric and quality of life outcomes. Here, we pinpoint (i) the communalities and differences of the brain‐injury induced affective disorder and MDD, (ii) the specific personalized needs regarding neurobiological, functional and psychiatric characteristics to improve affective, cognitive and sleep burden during neurorehabilitation, and (iii) future digital solutions to enable effective, scalable, and patient‐centered advances for better long‐term outcomes as well as good quality of life for our neuropsychiatric patients during and beyond neurorehabilitation.

**Disclosure:** Nothing to disclose.

## FW16_4

### Network‐targeted brain stimulation for neurorehabilitation of cognition and behaviour

#### S. Filipović

##### Institute for Medical Research, University of Belgrade, Serbia

Non‐invasive brain stimulation (NIBS) has been increasingly studied as a tool to promote neurorehabilitation for cognitive and behavioural impairments. A considerable body of evidence suggests that NIBS can have a beneficial effect, especially when coupled with other rehabilitation approaches. However, although encouraging, the results so far have not been unequivocal. One reason for the apparent lack of more substantial effects is the dominant single‐target focus in most clinical studies. NIBS techniques (such as transcranial magnetic stimulation – TMS, or transcranial direct current stimulation – tDCS) were typically applied over either the injured area or the homologue (contralateral) healthy area, or, in the case of cognitive impairments, over the dorsolateral prefrontal cortex (DLPFC). However, it is increasingly recognised that this approach is not effective enough to reset physiological mechanisms into a sustainable and robust recovery mode. Injury‐ or disease‐induced structural changes, beyond a loss of function directly associated with the lesion site(s), also disrupt large‐scale distributed brain networks (such as the default mode, salience, central executive, and sensorimotor). These disruptions can be a significant factor behind symptoms and impairments, but, even more importantly, they often prevent the recovery of functions, whether by restoration or by compensation. There is a growing understanding that individualised multi‐locus targeting, guided by functional and structural connectivity determined by functional imaging techniques (e.g., fMRI, DTI, EEG), can provide a breakthrough towards meaningful and enduring clinical applications.

**Disclosure:** Nothing to disclose.

## Vaccines technologies to protect brain health

## FW17_1

### Vaccines to prevent neurological infectious disease

#### A. Chaudhuri

##### Queen's Hospital, Department of Neurology, Glasgow, United Kingdom

## FW17_2

### Vaccine hesitancy and return of old neurological disease

#### E. Schmutzhard

##### Medizinische Universität Innsbruck, Universitätsklinik für Neurologie, Department of Neurology, Innsbruck, Austria

## FW17_3

### Vaccines to prevent and treat non‐infectious neurological disease

#### B. Willekens

##### Antwerp University Hospital and University of Antwerp, Department of Neurology, Edegem, Belgium

